# Diagnosis of coinfection by schistosomiasis and viral hepatitis B or C using ^1^H NMR-based metabonomics

**DOI:** 10.1371/journal.pone.0182196

**Published:** 2017-08-01

**Authors:** Liana Ribeiro Gouveia, Joelma Carvalho Santos, Ronaldo Dionísio Silva, Andrea Dória Batista, Ana Lúcia Coutinho Domingues, Edmundo Pessoa de Almeida Lopes, Ricardo Oliveira Silva

**Affiliations:** 1 Postgraduate Program in Chemistry, Fundamental Chemistry Department, Center for Exact and Natural Sciences, Universidade Federal de Pernambuco (UFPE), Recife, Pernambuco, Brazil; 2 Postgraduate Program in Tropical Medicine, Center for Health Sciences, Universidade Federal de Pernambuco (UFPE), Recife, Pernambuco, Brazil; 3 Department of Internal Medicine, Hospital das Clínicas, Universidade Federal de Pernambuco (UFPE), Recife, Pernambuco, Brazil; George Washington University, UNITED STATES

## Abstract

**Background:**

Diagnosis of liver involvement due to schistosomiasis in asymptomatic patients from endemic areas previously diagnosed with chronic hepatitis B (HBV) or C (HCV) and periportal fibrosis is challenging. H-1 Nuclear Magnetic Resonance (NMR)-based metabonomics strategy is a powerful tool for providing a profile of endogenous metabolites of low molecular weight in biofluids in a non-invasive way. The aim of this study was to diagnose periportal fibrosis due to schistosomiasis mansoni in patients with chronic HBV or HCV infection through NMR-based metabonomics models.

**Methodology/Principal findings:**

The study included 40 patients divided into two groups: (i) 18 coinfected patients with schistosomiasis mansoni and HBV or HCV; and (ii) 22 HBV or HCV monoinfected patients. The serum samples were analyzed through H-1 NMR spectroscopy and the models were based on Principal Component Analysis (PCA) and Partial Least Squares—Discriminant Analysis (PLS-DA). Ultrasonography examination was used to ascertain the diagnosis of periportal fibrosis. Exploratory analysis showed a clear separation between coinfected and monoinfected samples. The supervised model built from PLS-DA showed accuracy, R^2^ and Q^2^ values equal to 100%, 98.1% and 97.5%, respectively. According to the variable importance in the projection plot, lactate serum levels were higher in the coinfected group, while the signals attributed to HDL serum cholesterol were more intense in the monoinfected group.

**Conclusions/Significance:**

The metabonomics models constructed in this study are promising as an alternative tool for diagnosis of periportal fibrosis by schistosomiasis in patients with chronic HBV or HCV infection from endemic areas for *Schistosoma mansoni*.

## Introduction

Schistosomiasis is a widespread disease in the developing world and the most important water-based disease of tropical zone [1]. This neglected disease affects 200 million people in 78 countries, mainly across Africa, Asia and South America, and remains a major public health problem [2]. In Brazil, *Schistosoma mansoni* has infected approximately 12 million persons, mostly in the Northeastern region [3].

Schistosomiasis mansoni (SM) affects the mesenteric portal venous system with a range of clinical manifestations, from asymptomatic to extremely severe disease. Although, about 40% of infected patients are asymptomatic and only 10% have a more severe disease [4]. In the liver, the pathogenesis of SM is related to the granuloma formation around the eggs, leading to periportal fibrosis (Symmers’ fibrosis) and subsequent portal hypertension, splenomegaly and esophageal varices [5]. The rupture of esophageal varices may promote digestive hemorrhage requiring blood transfusion that would spread hepatic virus.

Data on schistosomiasis, hepatitis B virus (HBV) or hepatitis C virus (HCV) coinfection are inconsistent [6]. It is known that the coinfection can aggravate or accelerate the evolution of fibrosis, and a high prevalence of the coinfection is often observed in countries where SM is endemic [5,7].

Schistosomiasis infections are diagnosed by a clinical history of contact with a fresh water source, such as rivers or streams from endemic areas, followed by the detection of eggs in the stool [8]. Moreover, the presence of periportal fibrosis in mainly established by imaging methods such as ultrasonography (US), CT scan and MR scan [2]. US is considered as the primary modality of choice and plays an integral role in the diagnosis of liver involvement in schistosomiasis, since it can show fibrosis around the periportal spaces, splenomegaly, enhanced portal vein dimensions, and the presence of collateral vessels [5,9]. However, US is a subjective procedure, examiner-dependent and the liver parenchymal heterogeneity caused by viral infection might hamper visualization of periportal fibrosis in some patients [10].

Since the coinfection of schistosomiasis with HBV or HCV can aggravate the evolution of fibrosis, the diagnosis of coinfection is crucial to evaluate the severity of the disease process and its complications [2]. Hence, there is a need for more accurate and sensitive diagnostic techniques for periportal fibrosis, mainly for asymptomatic chronic schistosomiasis coinfected patients.

As an alternative, the Nuclear Magnetic Resonance (NMR)-based metabonomics strategy has proved its value in disease diagnosis. This strategy is often used when there is interest in classifying samples by their biochemical status in response to diseases [11,12], since NMR spectroscopy is a powerful tool for identifying changes in metabolite profiling as a result of changes in the biochemical status. A disadvantage is the fact that an NMR spectrometer is expensive. However, comparing to US, NMR is not a subjective technique. Moreover, NMR offers a non-invasive analysis and with only one serum sample it would be possible to find out with high accuracy if the patient has coinfection or not. The use of specific statistical tools for an efficient interpretation of NMR spectra is necessary, due to complexity of the process [13].

Many encouraging results have been obtained using NMR-based metabonomics to investigate diseases, including studies on hepatitis C [14], urological cancer [15] and schistosomiasis [16,17].

Therefore, the aim of this study was to diagnose periportal fibrosis due to schistosomiasis mansoni in patients with chronic HBV or HCV infection through ^1^H NMR-based metabonomics models.

## Patients and methods

### Patient characteristics

The study included 40 patients aged between 21 and 82 years attending the Hepatology outpatient clinic at the Hospital das Clínicas of Universidade Federal de Pernambuco (HC-UFPE) from October 2012 to May 2016. They were stratified into two groups: 18 coinfected patients with SM and HBV or HCV chronic infection and 22 HBV or HCV chronic monoinfected patients. Among the coinfected group, 7 had HBV infection (38.9%) and 11 HCV (61.1%). Among the monoinfected group, 17 had HBV infection (77.3%) and 5 HCV (22.7%).

The diagnosis of schistosomiasis mansoni was based on the clinical history of contact with fresh water sources from endemic areas and reports of prior treatment for schistosomiasis. The diagnosis of periportal fibrosis was confirmed by US findings, along with hypertrophy of the left liver lobe and splenomegaly. US exams were performed by a single experienced operator using a Siemens Acuson X 150^®^ device with a 3.5 MHz convex transducer.

Hepatitis chronic infection was diagnosed by hepatitis B surface antigen (HBsAg) and total hepatitis B core antibody (anti-HBc) positive results or anti-HCV antibodies positive, for more than 6 months, detected by microparticle immunoassay (CMIA) by Architect (Abbott Diagnostics, USA) at the Central Laboratory of (HC-UFPE).

Patients receiving any antiviral treatment, ethanol consumption, HIV coinfection or undergoing immunosuppressive therapy were excluded from the study.

### Ethics statement

This study was approved by the Ethics Committee on Research Involving Human Subjects of the Health Sciences Center—Universidade Federal de Pernambuco (CCS-UFPE), Recife, Brazil (Approval no. 1.782.771/2016) and all patients were adults and signed terms of informed consent.

### Sample collection and laboratory analysis

Sera from all patients were collected and liver function tests including alanine and aspartate aminotransferase (ALT and AST), gamma-glutamyl transferase (GGT), alkaline phosphatase (ALP), and lipid profile (total cholesterol, HDL cholesterol, LDL cholesterol and triglycerides) were carried out using Wiener Lab^®^ kits in a Wiener Lab^®^ autoanalyzer (Wiener Lab Group, Argentina), according to the manufacturer’s instructions. Part of the sera was stored at minus 20°C for a short period of time until the NMR analysis could be performed.

Fibrosis stage for both groups was based on AST to Platelet Ratio Index (APRI) with a cutoff score of 1.5 for detection of significant fibrosis (F2 to F4 by METAVIR) [18]. In addition, periportal fibrosis on the coinfected group was based on US patterns in mild, moderate or advanced fibrosis, according to the World Health Organization's Niamey protocol [19], using a Siemens Acuson X 150^®^ with a 3.5 MHz convex transducer for all patients by the same researcher.

### Univariate statistical analysis

To investigate the distribution of demographic and clinical laboratory data between groups, univariate tests were performed using GraphPad Prism 6 software (GraphPad Software, Inc., La Jolla, CA) with unpaired Student's t-test, Mann-Whitney, Fisher’s exact or χ2 tests, as appropriate. A *p* value <0.05 was set as the level of statistical significance.

### NMR analysis

Serum samples were prepared by mixing 400 μl of serum and 200 μl of D_2_O, using the signal attribute to lactate (δ 1.33 ppm) as a chemical shift reference. All ^1^H NMR spectra were recorded in a Varian Unity Plus spectrometer operating at 300 MHz, using NMR tubes of 5 mm. After homogenization, the ^1^H NMR spectrum was acquired using a sequence of pulses with presaturation (PRESAT) of the water signal hyphenated to the Carr-Purcell-Meiboom-Gill (CPMG) pulse sequence, which was employed as a T2 filter. The following parameters were used: spectral window of 4.8 kHz, saturation delay of 2.0 s, acquisition time of 1.704 s, 90° RF pulse, temperature of 25°C, 88 cycles, tau equal to 0.0004 s, bigtau equal to 0.07 s and 128 scans. The line broadening used was 0.3 Hz. Baseline and phase distortions were corrected manually. Using the MestreNova 9.0 software, the region between δ 4.20 and 0.04 ppm was binned into 104 bins with 0.04 ppm-wide bins. One matrix was built with 40 rows (cases) and 105 variables (bins of the ^1^H NMR spectrum plus class variable), and then was submitted to multivariate analysis.

### Framework for chemometric analysis

Multivariate statistical treatment of spectra data allows a simple, fast and reliable analysis, using tools such as Principal Components Analysis (PCA), Partial Least Squares—Discriminant Analysis (PLS-DA) and Simple Classification Analysis (SIMCA) [20].

The models based on PCA and PLS-DA were constructed via the web-based platform for metabonomics studies, namely MetaboAnalyst 3.0 [21]. In the pre-processing step, each sample was normalized by sum (cumulative intensity of the spectrum). This was performed to compare the spectral data, avoiding problems with sample dilutions, for example [22]. The validation of the PLS-DA model was based on two methods: (i) the leave-one-out cross-validation method (LOOCV), where the optimal number of latent variables for the PLS-DA model was determined, thus providing the basis for the computation of the predictive ability (Q^2^), determination coefficient (R^2^), and the classification accuracy of the model; and (ii) the permutation test, which made 2000 permutations of the class label to verify the accuracy of the PLS-DA model.

The PLS-DA model also provided a quantitative measure of the discriminating power of each spectral bin. For this purpose, we made use of the variable importance in the projection (VIP) score. VIP is a weighted sum of squares of the PLS loadings. These weights are based on the amount of explained variance of the dependent variable in each PLS dimension. For deciding between discriminatory and non-discriminatory bins, a VIP score cut-off of 1 was used. Discriminatory signals were attribute to metabolites using HMDB platform and also based on the literature [23–26].

## Results

Table 1 displays the clinical and laboratory characteristics of the coinfected and monoinfected groups. There were no significant differences (p <0.05) between the two groups of patients, except for total serum cholesterol levels, which were slightly higher in the monoinfected group (p = 0.0421).

**Table 1:** Clinical and laboratory characteristics of the coinfected (n = 18) and monoinfected groups (n = 22).

Characteristics	Coinfected group (n = 18)	Monoinfected group (n = 22)	*p* value
Age (years)	53.7± 13.7	45.6± 15.2	0.0856^a^
Gender (n, %)			
Male	13(72.2%)	10(45.5%)	0.1159^b^
Female	5(27.8%)	12(54.5%)	
ALT (/ULN)*	1.13(0.70–1.70)	0.82(0.61–0.82)	0.2595^c^
AST (/ULN)*	0.96(0.63–1.60)	0.72(0.52–1.39)	0.3348^c^
GGT(/ULN)*	1.87(0.61–4.23)	1.01(0.66–1.41)	0.2162^c^
ALP (/ULN)*	1.57(1.09–2.20)	1.22(0.95–1.35)	0.0464^c^
Total cholesterol (mg/dL)	155± 30.29	179.2± 35.66	**0.0421**^a^
HDL (mg/dL)	47.67± 9.67	55.79± 14.85	0.0718^a^
LDL (mg/dL)	86.44± 33.17	100.7± 31.74	0.2094^a^
Triglycerides (mg/dL)*	93.35(71.9–134.9)	84.45(62.1–152.1)	0.7655^c^
APRI	0.68(0.63–2.59)	0.42(0.27–0.56)	0.0536^c^

^a^Unpaired *t* test;

^b^Fisher’s exact test;

^c^Mann-Whitney test.

ALT: Alanine aminotransferase; ALP: Alkaline phosphatase; APRI: Aspartate-to-platelet ratio; AST: Aspartate aminotransferase; GGT: Gamma-glutamyl transferase; HBV: Hepatitis B virus; HCV: Hepatitis C virus; HS: Hepatic schistosomiasis; HDL: high density lipoprotein; LDL: low density lipoprotein. ULN: upper limits of normal. Data presented as mean ± standard deviation except those marked with an asterisk (*), which are presented as median values (interquartile range in brackets).

Regarding the US patterns of periportal fibrosis in liver parenchyma due to SM infection, 9 patients showed mild (50%) and 4 moderate (22.2%) patterns, while 5 had advanced fibrosis (27.8%). Whereas, using APRI score to measure liver fibrosis, 8 patients (20%, 2 from the monoinfected group and 6 from the coinfected group) had significant fibrosis stage (F2 to F4), while 32 patients (80%) had METAVIR stage below F2. The median APRI score for the monoinfected group was 0.42, and 0.68 for the coinfected group.

**Fig 1:**
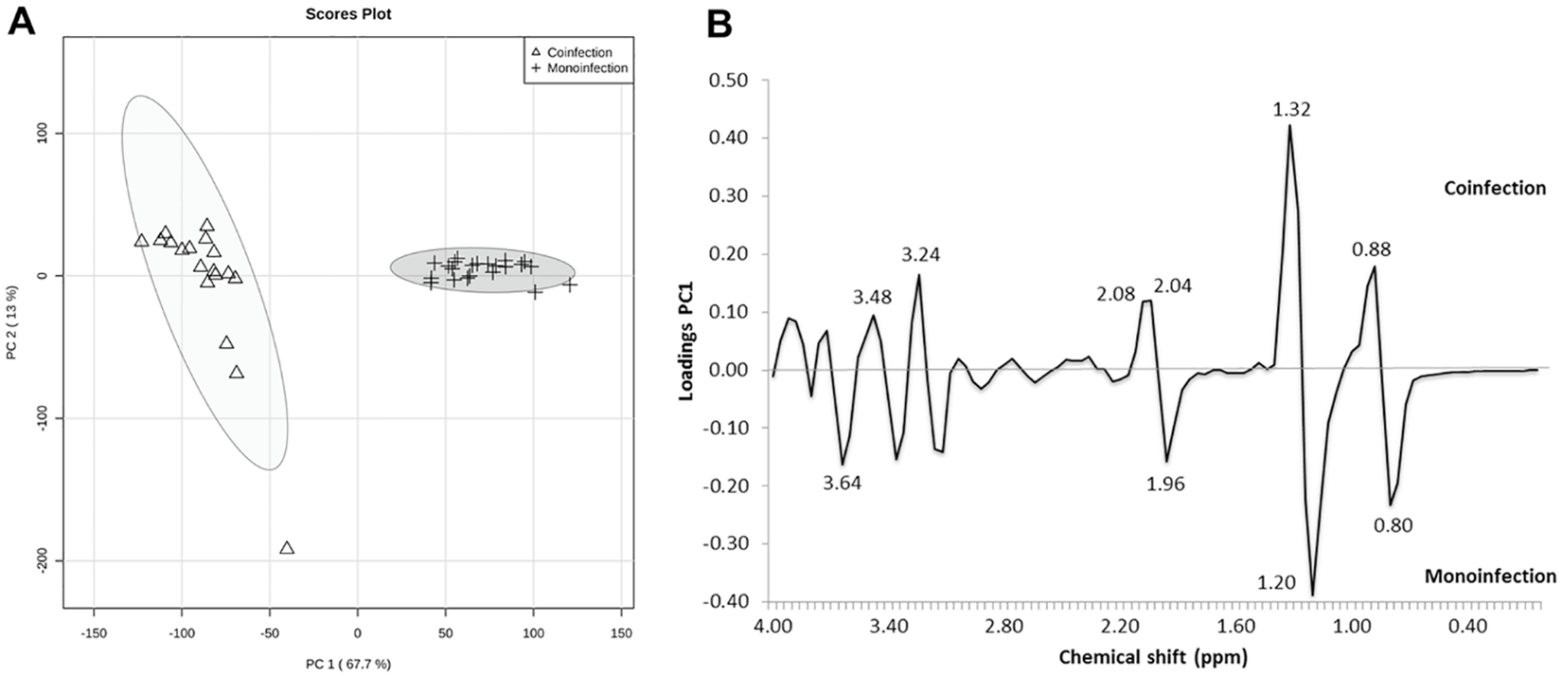
Results from PCA modeling. (A) PCA scores of coinfected patients and monoinfected patients. (B) Loading scatter plot of PC1 versus PC2, explaining 80.7% of variance.

**Fig 2:**
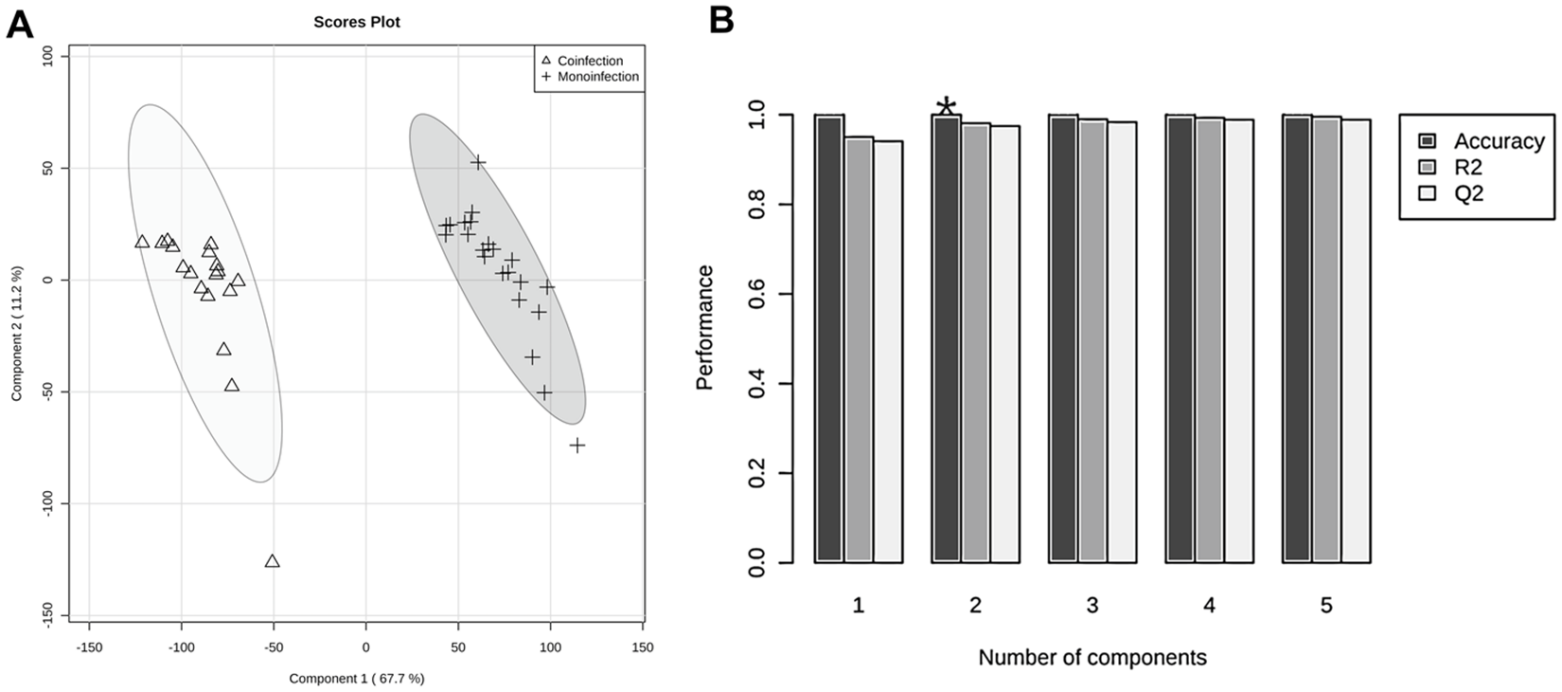
Results from PLS-DA modeling. (A) Scores scatter plot discriminating among coinfected and monoinfected patients. (B) The optimal number of PLS-DA components, according to the squared correlation coefficient (R^2^), the predictive ability (Q^2^), and the accuracy of the model; the asterisk indicates the best number of components based on accuracy of the model.

**Fig 3:**
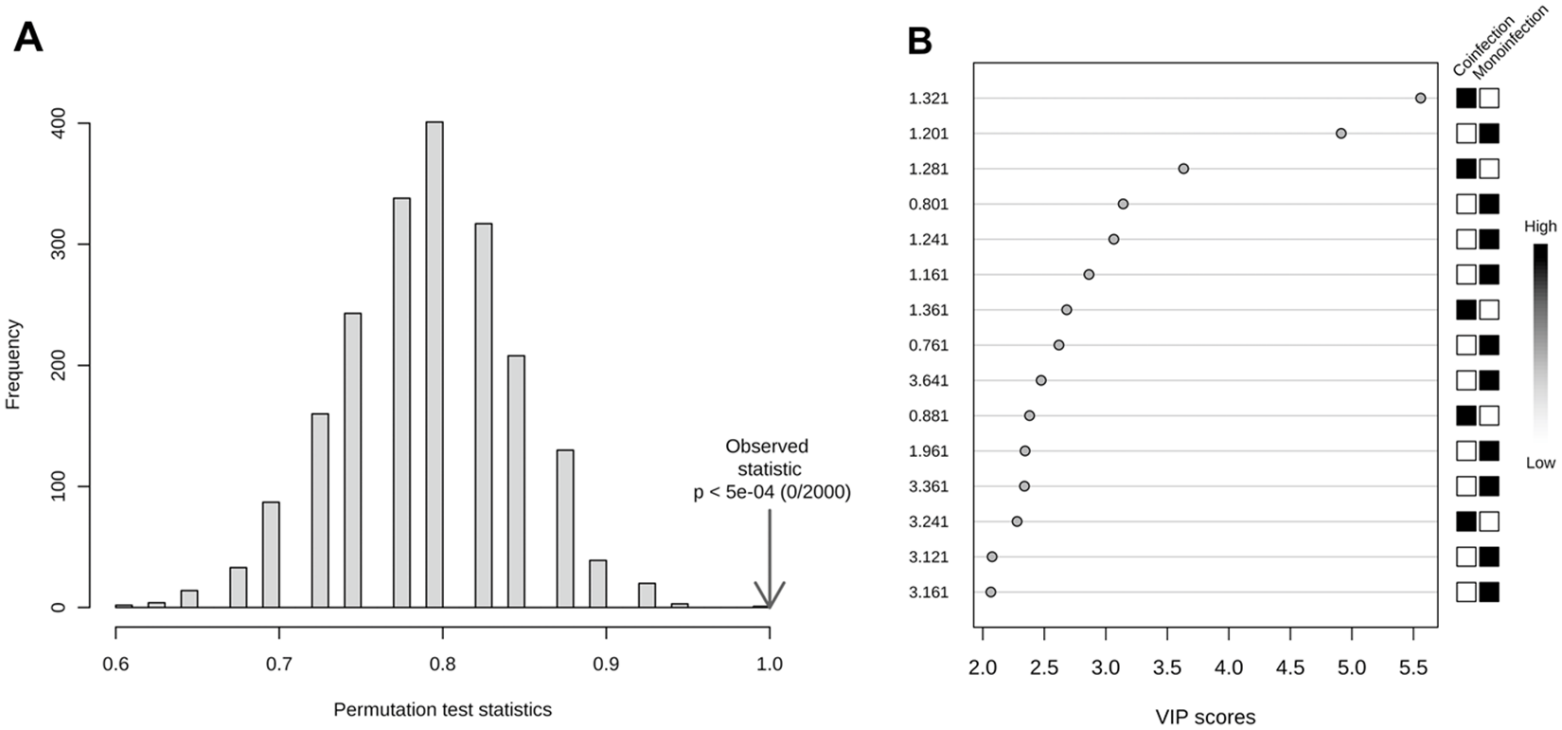
Results from PLS-DA modeling. (A) Permutation test statistic at 2000 permutations with observed statistic of the model prediction accuracy with *p* < 0.0005. (B) VIP scores from the metabonomics model using two components. The boxes on the right represent the relative integral of the corresponding bin in each group (monoinfection and coinfection).

Fig 1 exhibits the results obtained from PCA. PC1 and PC2 explain 80.7% of the variation in the data. The modeling phase for the two groups (coinfected and monoinfected groups) was based on the PLS-DA formalism (Fig 2). This model was cross-validated, resulting in accuracy, R^2^ and Q^2^ values equal to 100%, 98.1% and 97.5%, respectively (Fig 2B). As well, we undertook an alternative way of model validation, using permutation analysis involving 2000 permutations of the class label and computation of the accuracy of the PLS-DA model with two components (as indicated in the LOOCV-based analysis), and the result was *p* < 0.0005 (Fig 3A).

**Fig 4:**
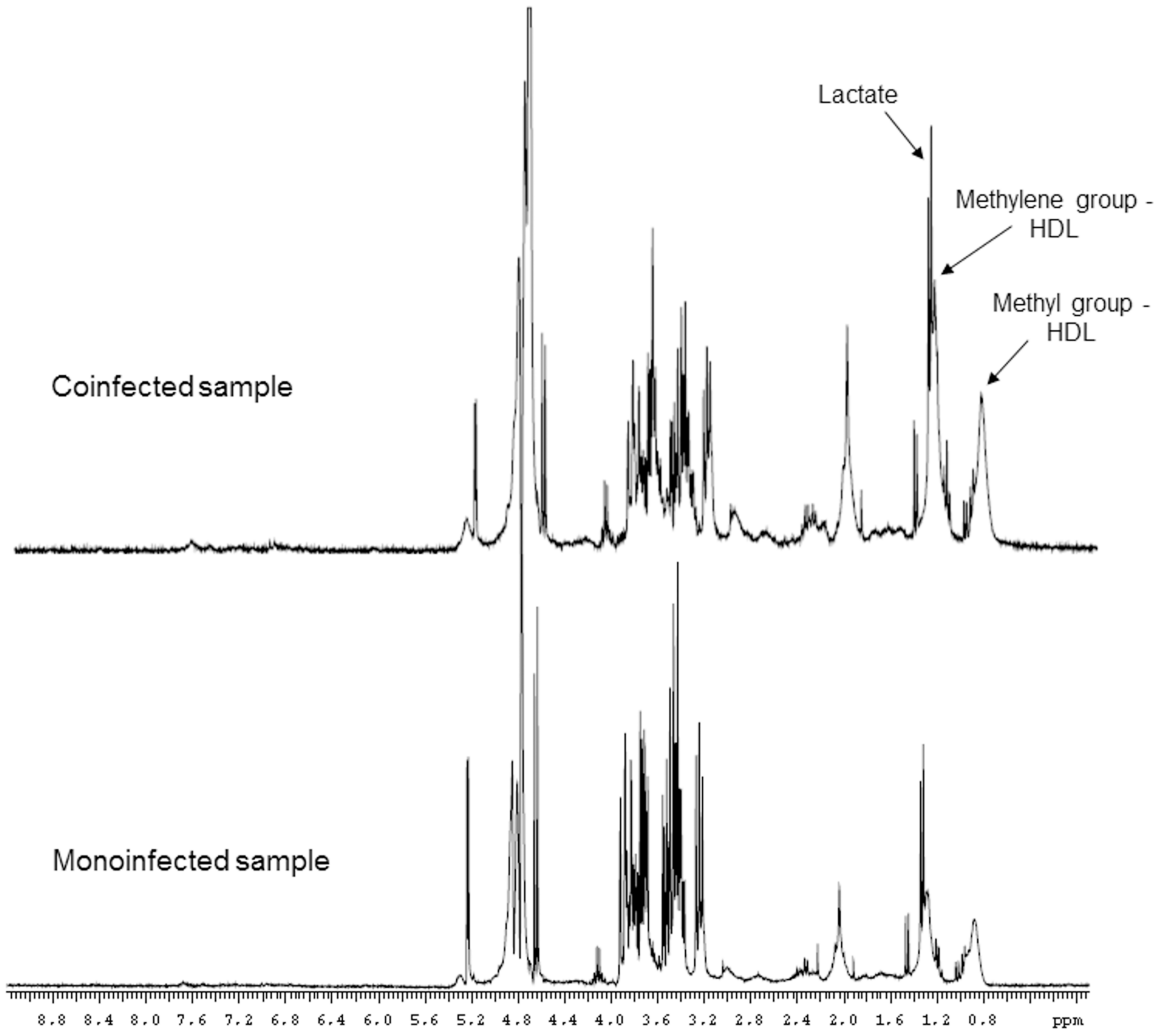
^1^H NMR spectra (PRESAT-CPMG, 300 MHz) of serum, with attribution of signals responsible for discrimination.

In the VIP score plot (Fig 3B), the two bins that best distinguished the samples were δ 1.32 and 1.20 ppm. These chemical shifts had been previously identified by visual inspection of the PCA loadings plot (Fig 1B). Considering a VIP value greater than 2.5, we identified three spectral regions responsible for the discrimination: (1) δ 1.28–1.36 ppm, attributed to methyl group from lactate; (2) δ 1.16–1.24 ppm, attributed to methylene groups from HDL cholesterol; and (3) δ 0.76–0.80 ppm, attributed to methyl groups of HDL cholesterol [27]. According to the VIP plot, the lactate serum levels were higher in the coinfected group, while the signals attributed to HDL cholesterol were more intense in the monoinfected group. Fig 4 shows ^1^H PRESAT-CPMG NMR spectra of serum samples, with indication of spectral regions used in the metabonomics modeling.

## Discussion

No statistically significant differences were found in the clinical and laboratory characteristics between the two groups of patients, except for total serum cholesterol levels.

In this primary study, most patients in the coinfected group (72.2%) showed mild/moderate periportal fibrosis in their US, making it difficult to differentiate between normal liver and other diseases [28]. In addition, in both groups there was a predominance of non-significant parenchymal fibrosis due to chronic viral hepatitis (METAVIR <F2).

In fact, diagnosis of liver involvement due to chronic SM in coinfected patients with mild/moderate periportal fibrosis poses more challenges, since the US evaluation is more difficult due to the presence of parenchymal fibrosis induced by the virus, when compared to patients with advanced periportal fibrosis. Moreover, in this study only one third of coinfected patients presented advanced periportal fibrosis, making the diagnosis more demanding. In clinical practice, these data show the meaning of the metabonomics models developed in our study.

As shown in Fig 1, the PCA results indicates that there is a natural grouping among the samples, discriminating between monoinfected patients and those coinfected with SM. Fig 1A exhibits the relationship between the scores in PC1 and PC2, showing that the samples were only discriminated by PC1. The main discriminatory bins are ranged from δ 1.16 ppm to 1.36 ppm, being the signal centered at δ 1.20 ppm (δ 1.16–1.24 ppm), associated with the monoinfected group, while the signal centered at δ 1.32 ppm (δ 1.28–1.36 ppm) is associated with the coinfected group. In fact, this assumption was also supported by the VIP score study as can be seen later in this section.

Similar to the exploratory PCA, PLS-DA makes it possible to see the significant difference between the groups of coinfected patients and those monoinfected. The results of the PLS-DA model and its cross-validation show that the model is accurate and has a promising predictive performance for discriminating between coinfected and monoinfected patients. In the permutation analysis involving 2000 permutations of the class label, the *p* <0.0005 indicates that the accuracy of a permuted PLS-DA model would rarely be better than the one of the model adjusted to the original data.

In fact, Wang et al., using a *S*. *mansoni*–mouse model based on ^1^H NMR spectroscopy, reported that the lactate concentration varied between animals but was not found to be significant in discriminating between infected and control mice. Moreover, the metabolic signature of an *S*. *mansoni* infection consisted of reduced levels of the tricarboxylic acid cycle intermediates, including citrate, succinate, and 2-oxoglutarate, and increased levels of pyruvate, suggesting stimulated glycolysis [16,29]. Although, the consequences of glucose losses and the local elevation of lactate levels in the bloodstream depend on the severity of the infection and nutritional status of the host [17]. In addition, it is reported that in liver diseases, the aerobic metabolism is blocked, resulting in glycolysis and pentose phosphate pathway becoming the main glucose metabolic pathways. Glycolysis could produce large amounts of lactic acid, leading to a high lactic acid level [30].

There are reports in the literature that associate advanced fibrosis [31] and cirrhosis [30] with lower levels of LDL and HDL. In addition, schistosomiasis patients are reported to have dyslipidemia resulting in reduced total cholesterol, LDL and triglycerides when compared to healthy individuals, which depend on apolipoprotein E gene polymorphism [32].

In this study, we found that total cholesterol serum levels were lower in coinfected patients when compared to the monoinfected group. Although not statistically significant, there was a tendency for lower values of HDL and LDL and higher values of triglycerides in the coinfected group, which may explain the importance of signals attributed to HDL in the discrimination among the samples. Perhaps, the number of patients in our study was not sufficient to reveal differences that would be more significant.

It is interesting to note that the metabonomics models indicated differences between the groups regarding the serum levels of triglycerides and HDL. However, these differences were minimal and not observed in the serum levels measurement by conventional assays.

Furthermore, ^1^H NMR-based metabonomics has been used successfully to discriminated patients with HCV infection with high sensitivity and specificity [14]; to distinguish *S*. *mansoni*-infected from uninfected individuals before and after chemotherapy with praziquantel [17]; and to characterize metabolic profiles of liver cirrhosis and hepatocellular carcinoma [33]. These results suggest that NMR spectra combined with pattern recognition analysis techniques offer a great benefit to early diagnosis of human diseases.

In summary, this paper has been an attempt to provide a framework for the discrimination between coinfected patients with chronic SM and viral hepatitis. The proposed approach was based on exploratory PCA and subsequent PLS-DA of pre-processed binned ^1^H NMR spectra of serum samples. Exploratory PCA for ^1^H NMR data showed a clear separation between coinfected and monoinfected samples. The supervised model built using PLS-DA showed a predictive ability of 100% accuracy, indicating that the model can be useful for classification of serum samples of patients without prior knowledge of the condition (coinfection with SM or only HBV or HCV). The spectral regions responsible for discrimination were attributed to lactate (δ 1.32 ppm) and HDL (broad signals at δ 0.76–0.80 ppm and δ 1.16–1.24 ppm).

Therefore, this pilot study shows the potential for these metabonomics models to be used as an alternative to conventional methods to confirm the diagnosis of periportal fibrosis by schistosomiasis in patients with chronic HBV or HCV infection from endemic areas for *S*. *mansoni*.
